# Association Between Helicobacter pylori Infection and Iron Deficiency Anemia in Adult Patients Attending a Tertiary Care Hospital

**DOI:** 10.7759/cureus.111431

**Published:** 2026-06-24

**Authors:** Ajmal Khan, Syeda Mah Noor Fatima, Sheikh Mudassir Mukhtar Ahmad Vohra, Jawad Ahmad, Haris Ashfaq, Shehla Gul, Laraib Fatima, Adnan Afzal, Tamanna Nazir, Dur E Nayab, Sumaria Nazir, Naqeeb Ullah

**Affiliations:** 1 Medicine, Mardan Medical Complex, Mardan, PAK; 2 Emergency Medicine, Maroof Healthcare Services Pvt Ltd, Islamabad, PAK; 3 Medicine, Barnsley Hospital NHS Foundation Trust, Barnsley, GBR; 4 Neurology, Lady Reading Hospital, Peshawar, PAK; 5 Medicine, Lady Reading Hospital, Peshawar, PAK; 6 Medical School, Gomal Medical College, Dera Ismail Khan, PAK; 7 Obstetrics and Gynaecology, Hayatabad Medical Complex, Peshawar, PAK; 8 Orthopaedics and Trauma, Rehman Medical Institution, Peshawar, PAK; 9 Internal Medicine, Hayatabad Medical Complex, Peshawar, PAK; 10 Internal Medicine, Lady Reading Hospital, Peshawar, PAK; 11 Public Health, Cardiff University, Cardiff, GBR

**Keywords:** ferritin, helicobacter pylori, hemoglobin, iron deficiency anemia, mean corpuscular volume (mcv), total iron-binding capacity (tibc)

## Abstract

Background: Iron deficiency anemia (IDA) has been increasingly associated with Helicobacter pylori (*H. pylori*) infection, especially in developing countries.

Objective: This study aimed to evaluate the association between *H. pylori* infection and IDA severity and to compare hematological parameters between *H. pylori*-positive and -negative adult patients attending a tertiary care hospital.

Methodology: This was a cross-sectional analytical study conducted at Lady Reading Hospital, Peshawar, from January 2025 to December 2025. Consecutive non-probability sampling was used to recruit a total of 236 adult patients with IDA. Stool antigen testing was used to diagnose infection with *H. pylori*. Hematological parameters, such as hemoglobin, serum ferritin, serum iron, total iron-binding capacity (TIBC), and mean corpuscular volume (MCV), were determined. Data were analyzed using IBM SPSS Statistics, version 26, while the chi-square test and independent t-test were used to determine the association of the data.

Results: *H. pylori* infection was positive in 136 (57.63%) patients and negative in 100 (42.37%). A significant association was observed between infection and moderate-to-severe anemia (p < 0.001, odds ratio (OR) = 2.58). Hemoglobin levels were significantly lower in infected patients (9.31 ± 1.42 vs. 10.72 ± 1.39 g/dL, p < 0.001). Similarly, serum ferritin (11.02 ± 5.10 vs. 19.48 ± 6.25 ng/mL, p < 0.001), serum iron (40.65 ± 11.02 vs. 59.37 ± 12.10 µg/dL, p < 0.001), TIBC (415.28 ± 39.21 vs. 354.62 ± 40.85 µg/dL, p < 0.001), and MCV (70.84 ± 6.72 vs. 78.91 ± 6.88 fL, p < 0.001) also showed statistically significant differences between groups.

Conclusion: IDA severity and hematologic parameters are significantly associated with *H. pylori *infection in adult patients.

## Introduction

Iron deficiency anemia (IDA) is a very common form of nutritional and haematological disorders in developed and developing countries [1]. It is associated with diminished synthesis of hemoglobin due to inadequate iron supply, leading to impaired oxygen-transport capacity of the blood [2]. Patients with IDA typically experience fatigue and general weakness, as well as dizziness, palpitations, decreased exercise tolerance, and diminished quality of life [3]. Malnutrition, chronic infections, gastrointestinal (GI) disorders, and reduced access to healthcare services are all important aspects that contribute to the burden of IDA in low- and middle-income countries [4]. While poor iron nutrition and chronic blood loss are the two main causes, the involvement of infectious and inflammatory diseases, which affect iron absorption, utilization, and metabolism, has been increasingly recognized [5].

*Helicobacter pylori *(*H. pylori*) is a gram-negative bacterium that is spiral-shaped, microaerophilic (grows in the absence of oxygen), and colonizes the gastric mucosa and infects almost half the world's population [6]. In developing nations, *H. pylori *infection is far more common, and this is believed to be due to the fact that the developing country also suffers from overcrowding, lack of sanitation, and low socioeconomic status [7]. It is classically described with chronic gastritis, peptic ulcer disease, gastric mucosal atrophy, and gastric malignancies. However, it has increasingly been postulated that it plays a role in extra-gastric manifestations, especially IDA [8].

Several biological mechanisms have been suggested to account for this association, such as chronic gastric inflammation causing a change in iron absorption, decreased level of gastric acid secretion, thereby decreasing the solubility of iron, micro-bleeding in the GI tract, and competitive use of host iron by the organism for its survival and multiplication [9,10]. Moreover, because of the possibility of continued infection, there can be a reduction in the mobilization of iron stores, which can contribute to anemia development [11].

The primary objective of this study was to evaluate the relationship between *H. pylori* infection and the severity of IDA, along with associated hematological parameters in adult patients presenting to a tertiary care hospital. The secondary objective was to compare hematological indices, including hemoglobin, serum ferritin, serum iron, total iron-binding capacity (TIBC), and mean corpuscular volume (MCV), between *H. pylori*-positive and *H. pylori*-negative patients with IDA.

The eradication of *H. pylori *has been shown in interventional studies to improve hemoglobin and serum ferritin levels, suggesting a possible causal link between *H. pylori* infection and IDA [12,13]. Despite growing evidence, this association is not yet routinely recognized in clinical practice, particularly among adults in tertiary care settings. This relationship is clinically important for early detection and targeted management strategies in high-prevalence countries such as Pakistan.

## Materials and methods

Study design and setting

This study was an analytical cross-sectional study carried out in Lady Reading Hospital, Peshawar, which is a large tertiary care referral hospital. All the participants included were diagnosed cases of IDA. Adult patients who presented to the outpatient and inpatient departments with clinical and laboratory characteristics compatible with the diagnosis of IDA were included in the study and had their *H. pylori* status determined.

Study duration

The study was carried out for one year from January 2025 to December 2025.

Sample size and sampling technique

The sample size for this study was calculated using the World Health Organization sample size formula for cross-sectional studies (n = Z² × P × [1 − P] / d²), where n is the required sample size, Z is the standard normal deviate corresponding to a 95% confidence interval (1.96), P is the expected prevalence of IDA (assumed as 0.5 to yield the maximum sample size), and d is the margin of error set at 7%. Using this approach, the minimum required sample size was calculated to be approximately 196, which is consistent with the methodology applied in a recent similar study by Khan et al., investigating iron deficiency among *H. pylori*-infected patients [14]. To account for potential non-response and incomplete data, the sample size was rounded to 200 participants; however, during the study period, a total of 236 eligible patients were ultimately enrolled through consecutive sampling to ensure adequate statistical power and robustness of results.

Inclusion and Exclusion Criteria

IDA was defined as anemia, as defined by hematologic and biochemical criteria, and was included in this study only in adult patients ≥18 years of age who presented with anemia prior to enrollment. All participants gave written informed consent before being enrolled. All patients were screened with standardized diagnostic testing for *H. pylori *infection after enrollment to determine whether the patients were *H. pylori* positive or negative. Anemia patients caused by other reasons than iron deficiency, such as hemolytic anemia, aplastic anemia, and thalassemia patients, were excluded. Also, patients with chronic liver disease, chronic kidney disease, malignancy, blood transfusion, or iron supplementation within the previous three months before the study began were excluded. Patients receiving *H. pylori *eradication, proton pump inhibitors, antibiotics, or bismuth compounds within four weeks before enrollment were also excluded. Pregnant women were not included in the study. The detailed inclusion and exclusion criteria used for participant selection are presented in Table 1.

**Table 1:** Inclusion and exclusion criteria of the study participants Acid-suppressing and related medications included proton pump inhibitors (PPIs), H2 receptor antagonists (e.g., ranitidine, famotidine), potassium-competitive acid blockers (e.g., vonoprazan), antibiotics, bismuth compounds, and other commonly used over-the-counter acid-reducing agents. These were excluded due to their potential to interfere with the *H. pylori *stool antigen test accuracy.

Inclusion criteria	Exclusion criteria
Adult patients aged ≥18 years	Hemolytic anemia
Diagnosed cases of iron deficiency anemia (IDA)	Aplastic anemia
Hemoglobin <13 g/dL in males and <12 g/dL in females	Thalassemia
Low mean corpuscular volume (MCV)	Chronic liver disease
Low serum iron levels	Chronic kidney disease
Elevated total iron binding capacity (TIBC)	Malignancy
Serum ferritin <15 ng/mL	Blood transfusion within the previous three months
Patients presenting to the outpatient or inpatient departments of Lady Reading Hospital	Iron supplementation within the previous three months
Provided written informed consent	Receiving H. pylori eradication therapy within four weeks before enrollment
	Use of proton pump inhibitors within four weeks before enrollment
	Use of antibiotics within four weeks before enrollment
	Use of bismuth compounds within four weeks before enrollment
	Pregnancy
	Clinically diagnosed peptic ulcer disease or erosive gastritis with evidence of active or recent gastrointestinal bleeding
	Known gynecological causes of chronic blood loss (e.g., menorrhagia, uterine fibroids, abnormal uterine bleeding disorders)

Data collection

Detailed demographic and clinical data, including age, gender, socioeconomic status, dietary habits, GI complaints, and relevant medical history, were collected after obtaining written informed consent using a structured proforma. Venous blood samples were drawn under aseptic conditions and analyzed for complete blood count and iron profile, including hemoglobin, serum ferritin, serum iron, total iron-binding capacity (TIBC), and mean corpuscular volume (MCV), following standard laboratory protocols in the hospital diagnostic laboratory.

All participants were evaluated for *H. pylori *infection using validated diagnostic methods. Dietary iron intake was assessed at the time of enrollment using a structured dietary recall questionnaire administered by the investigators. Participants were asked about their habitual consumption of iron-rich foods, including red meat, poultry, fish, legumes, green leafy vegetables, fortified cereals, and other locally available dietary sources of iron during the preceding month. Based on the reported frequency and quantity of consumption, estimated dietary iron intake was categorized relative to age- and sex-specific recommended dietary allowances (RDA). Participants whose estimated intake was below the recommended daily requirement were classified as having low dietary iron intake, whereas those meeting or exceeding the recommended daily requirement were classified as having adequate dietary iron intake. This variable was included to account for dietary factors that could potentially influence iron status and act as confounders in the association between *H. pylori* infection and IDA.

Nonsteroidal anti-inflammatory drug (NSAID) use was defined as regular consumption (≥2 times per week for at least one month prior to enrollment). All hematological and iron profile parameters were obtained at the time of enrollment and prior to *H. pylori *testing, ensuring contemporaneous assessment of infection status and biochemical indices under standardized baseline conditions. GI symptoms were recorded as present if patients reported dyspepsia, epigastric pain, bloating, nausea, or early satiety persisting for ≥2 weeks prior to presentation.

Diagnosis of *H. pylori* infection

Stool antigen testing (HpSA) was used as the primary diagnostic modality for the detection of *H. pylori* infection due to its high sensitivity and specificity for active infection. In selected cases where endoscopy was clinically indicated, rapid urease testing (RUT) was performed as a confirmatory test. Patients were categorized as *H. pylori* positive only when stool antigen testing was positive; no indeterminate category was used in analysis to maintain diagnostic consistency. Endoscopic evaluation was not routinely performed and was reserved only for patients presenting with alarm features or strong clinical indications, primarily to exclude alternative upper GI pathology when necessary.

Assessment of IDA

IDA was diagnosed based on hematological and biochemical parameters, including reduced hemoglobin, low MCV, low serum iron, elevated TIBC, and decreased serum ferritin levels. Anemia was defined according to the WHO criteria as hemoglobin <13 g/dL in males and <12 g/dL in females, while iron deficiency was defined as serum ferritin <15 ng/mL. Severity of anemia was adapted for adult populations: mild (Hb 10-12.9 g/dL in males, 10-11.9 g/dL in females), moderate (8-9.9 g/dL), and severe (<8 g/dL) [3]. This standardized classification has been widely used in clinical research evaluating anemia severity and its associated risk factors.

Study variables

*H. pylori *infection (*H. pylori* + or -) was the independent variable, and IDA status was the dependent variable. Other factors were age, sex, body mass index (BMI), socioeconomic status, GI symptoms, dietary habits, hemoglobin, serum ferritin, serum iron, total iron-binding capacity, and mean corpuscular volume.

Statistical analysis

The data were analyzed using IBM SPSS Statistics for Windows, version 26.0 (released 2019, IBM Corp., Armonk, NY). Continuous variables were assessed for normality and presented as mean ± standard deviation (SD), while categorical variables were expressed as frequencies and percentages. The independent sample t-test was used to compare mean hematological parameters (hemoglobin, serum ferritin, serum iron, TIBC, and MCV) between *H. pylori*-positive and *H. pylori*-negative groups. The Chi-square test was applied to assess the association between *H. pylori *infection status and the severity of IDA, as well as other categorical variables. The strength of association between *H. pylori* infection and moderate-to-severe IDA was estimated using the odds ratio (OR) with 95% confidence intervals (CIs).

To reduce potential confounding and minimize bias inherent in cross-sectional design, key variables including age, gender, BMI, socioeconomic status, dietary iron intake, NSAID use, GI symptoms, and relevant exclusion criteria (such as chronic disease states and sources of blood loss) were evaluated during analysis. Where appropriate, stratified analyses were performed to assess the consistency of associations across subgroups. These steps were taken to improve internal validity and reduce the influence of confounding factors on the observed association. All statistical tests were two-tailed, and a p-value <0.05 was considered statistically significant.

Ethical considerations

The study was approved by the Lady Reading Hospital Institutional Review Board/Ethical Review Committee before the study began. All participants gave informed written consent (approval no. 1102/LRH/MTI (date: November 19, 2024)). Patient information was kept confidential and anonymous throughout the study. The study was carried out in compliance with the Declaration of Helsinki and the international ethical standards for human studies.

## Results

A total of 236 participants were included in the study. The study population demonstrated a higher proportion of middle-aged individuals, females, and participants from lower socioeconomic groups, reflecting a typical tertiary care hospital patient distribution. The majority were in the age group 31-45 years (84, 35.59%), followed by 18-30 years (62, 26.27%), 46-60 years (58, 24.58%), and >60 years (32, 13.56%), shown in Table 2. There were 106 (44.92%) males and 130 (55.08%) females. Regarding BMI, 38 (16.10%) were underweight, 122 (51.69%) had normal BMI, 56 (23.73%) were overweight, and 20 (8.47%) were obese. Most participants belonged to low socioeconomic status (140, 59.32%), followed by middle (74, 31.36%) and high (22, 9.32%). *H. pylori* infection was positive in 136 (57.63%) and negative in 100 (42.37%) patients. IDA severity was mild in 88 (37.29%), moderate in 102 (43.22%), and severe in 46 (19.49%) cases.

**Table 2:** Demographic characteristics, body mass index, H. pylori infection status, and severity of ida among study participants (n = 236) BMI: body mass index, IDA: iron deficiency anemia

Variable	Category	Frequency (n)	Percentage (%)
Age (years)	18–30	62	26.27
	31–45	84	35.59
	46–60	58	24.58
	>60	32	13.56
Gender	Male	106	44.92
	Female	130	55.08
BMI	Underweight	38	16.10
	Normal	122	51.69
	Overweight	56	23.73
	Obese	20	8.47
Socioeconomic status	Low	140	59.32
	Middle	74	31.36
	High	22	9.32
*H. pylori *status	Positive	136	57.63
	Negative	100	42.37
IDA severity	Mild	88	37.29
	Moderate	102	43.22
	Severe	46	19.49

The findings indicate a clear gradient of increasing anemia severity among patients with *H. pylori* infection, suggesting a strong association between infection status and disease burden. Table 3 shows a statistically significant association between *H. pylori* infection and severity of IDA (χ² = 14.21, df = 2, p = < 0.001). Among *H. pylori*-positive patients (n = 136), 38 (27.94%) had mild, 64 (47.06%) had moderate, and 34 (25.00%) had severe anemia, whereas in the *H. pylori*-negative group (n = 100), 50 (50.00%) had mild, 38 (38.00%) had moderate, and 12 (12.00%) had severe anemia. Overall, moderate-to-severe anemia was more frequent in infected patients (72.06%) compared to non-infected patients (50.00%). The odds of having moderate-to-severe IDA were significantly higher in *H. pylori*-positive patients (OR = 2.58, 95% CI: 1.52-4.38).

**Table 3:** Association between H. pylori infection and severity of iron deficiency anemia

*H. pylori* status	Mild n (%)	Moderate n (%)	Severe n (%)	Moderate–severe n (%)	Total	χ² (df)	p-value	OR (95% CI)
Positive	38 (27.94)	64 (47.06)	34 (25.00)	98 (72.06)	136	14.21 (2)	<0.001	2.58 (1.52–4.38)
Negative	50 (50.00)	38 (38.00)	12 (12.00)	50 (50.00)	100			

The hematological trends are consistent with more severe iron depletion and impaired iron metabolism in patients with *H. pylori* infection. Mean hemoglobin levels were significantly lower in *H. pylori*-positive patients (9.31 ± 1.42 g/dL) compared to *H. pylori*-negative patients (10.72 ± 1.39 g/dL, p < 0.001), shown in Table 4. Similarly, serum ferritin was reduced in the positive group (11.02 ± 5.10 ng/mL vs. 19.48 ± 6.25 ng/mL), serum iron was lower (40.65 ± 11.02 µg/dL vs. 59.37 ± 12.10 µg/dL), while TIBC was higher (415.28 ± 39.21 µg/dL vs. 354.62 ± 40.85 µg/dL) in *H. pylori*-positive patients. MCV was also significantly lower in infected patients (70.84 ± 6.72 fL vs. 78.91 ± 6.88 fL), with all comparisons showing statistically significant differences (p < 0.001).

**Table 4:** Hematological parameters by H. pylori status TIBC: total iron-binding capacity, MCV: mean corpuscular volume

Parameter	Positive (mean ± SD)	Negative (mean ± SD)	Mean difference	t-value	p-value
Hemoglobin (g/dL)	9.31 ± 1.42	10.72 ± 1.39	-1.41	7.63	<0.001
Serum ferritin (ng/mL)	11.02 ± 5.10	19.48 ± 6.25	-8.46	11.05	<0.001
Serum iron (µg/dL)	40.65 ± 11.02	59.37 ± 12.10	-18.72	12.18	<0.001
TIBC (µg/dL)	415.28 ± 39.21	354.62 ± 40.85	+60.66	11.89	<0.001
MCV (fL)	70.84 ± 6.72	78.91 ± 6.88	-8.07	9.92	<0.001

The distribution of clinical risk factors suggests that dietary insufficiency and GI symptoms are common among patients with IDA, while NSAID use represents a relevant but less frequent contributing factor. Dietary iron intake was low in 146 (61.86%) patients and adequate in 90 (38.14%), as shown in Table 5. NSAID use was reported in 98 (41.53%) patients, while 138 (58.47%) had no history of NSAID use. GI symptoms were present in 152 (64.41%) patients and absent in 84 (35.59%) patients.

**Table 5:** Clinical risk factors and their association with H. pylori infection / IDA NSAID: nonsteroidal anti-inflammatory drug, GI: gastrointestinal, IDA: iron deficiency anemia

Variable	Category	H. pylori positive n (%)	H. pylori negative n (%)	χ²-value	p-value	OR (95% CI)
Dietary iron intake	Low	98 (71.32)	48 (48.00)	13.87	<0.001	2.68 (1.55-4.63)
	Adequate	38 (28.68)	52 (52.00)			
NSAID use	Yes	64 (47.06)	34 (34.00)	4.07	0.044	1.72 (1.01-2.93)
	No	72 (52.94)	66 (66.00)			
GI symptoms	Present	102 (75.00)	50 (50.00)	16.21	<0.001	3.00 (1.76-5.11)
	Absent	34 (25.00)	50 (50.00)			

## Discussion

IDA has been increasingly associated with *H. pylori *infection, especially in resource-limited areas. In the current study, the prevalence of *H. pylori* infection was 57.63% (136/236) in IDA patients, which showed a high burden of *H. pylori* infection in the anemic population. This prevalence is similar to the reported 50-60% prevalence of *H. pylori* positivity in patients with IDA in a cross-sectional study in Pakistan, indicating a similar epidemiological pattern among IDA patients with similar socioeconomic status and hygiene among South Asians [15].

In the present study, the relationship between the severity of IDA and the *H. pylori* infection was statistically significant (χ² = 14.21, p ≤ 0.001). The OR for moderate-to-severe anemia was 2.58 (95% CI: 1.52-4.38) for *H. pylori*-positive patients compared to *H. pylori*-negative patients. The results are consistent with a systematic review and meta-analysis suggesting that *H. pylori *infection is a strong risk factor for anemia, with a risk that is increased by approximately 1.7 times in infected people [16]. This stronger correlation in the current study could be because diagnosis may have been delayed and background iron deficiency was higher among the local population.

Regarding hematological indices, the current study found significantly lower hemoglobin in infected patients (9.31 ± 1.42 g/dL vs. 10.72 ± 1.39 g/dL, p < 0.001) along with reduced serum ferritin (11.02 ± 5.10 ng/mL vs. 19.48 ± 6.25 ng/mL) and serum iron (40.65 ± 11.02 µg/dL vs. 59.37 ± 12.10 µg/dL). The present results are in line with the results of a meta-analysis study of RCTs, showing that *H. pylori* eradication is effective in significantly improving the hemoglobin and ferritin levels of IDA patients, indicating the causal role of *H. pylori* infection in iron metabolism impairment [17]. The uniformity of the observed decrease in iron stores in the various studies supports the biological plausibility of chronic infection causing iron depletion.

In addition, this study revealed that the total iron-binding capacity was increased (415.28 ± 39.21 vs. 354.62 ± 40.85 µg/dL) and MCV was decreased (70.84 ± 6.72 vs. 78.91 ± 6.88 fL) in infected patients, which suggests classical microcytic hypochromic anemia. In the same way, observational studies have shown that *H. pylori* infection leads to lower levels of ferritin and changed iron transport markers because of the diminished gastric acid secretion and lower iron absorption [16]. These hematological changes are in line with a hypothesis that chronic inflammation of the stomach is a factor in systemic iron depletion.

Several biological processes could account for the present observations: decreased gastric acid production, decreased iron solubility, and bacterial competition for host iron. Iron loss may also be exacerbated by chronic gastritis due to *H. pylori*, which may result in occult bleeding of the gastric mucosa. Experimental and clinical data suggest that *H. pylori* exploits host iron for its survival and thus worsens iron deficiencies in those who are susceptible [18,19].

Finally, dietary and behavioral factors of the present study add context to the relevance, since 61.86% of patients consumed a low amount of iron in their diet and 41.53% of patients reported having taken NSAIDs, all of which could be confounders or synergistic factors that could increase the severity of the anemia. Comparable findings have been noted in low-income populations that suffer from nutritional deficiency and chronic infection, interpreting the separate impacts of *H. pylori* infection on iron status [17,20].

Strengths and limitations

The study has several strengths. A key strength is the use of clearly defined hematological and biochemical criteria for the diagnosis of IDA, including hemoglobin, serum ferritin, serum iron, TIBC, and MCV, which enhances diagnostic accuracy. In addition, stool antigen testing was used as a validated, non-invasive, and cost-effective method for the detection of *H. pylori* infection, improving feasibility and diagnostic reliability in a resource-limited setting. The study also evaluated anemia severity and multiple hematological indices, allowing a comprehensive assessment of iron status in relation to infection.

However, several limitations should be acknowledged. The cross-sectional design limits the ability to establish a causal relationship between *H. pylori* infection and IDA. The study was conducted at a single tertiary care hospital, which may limit generalizability to the broader population. Although important potential confounders, such as dietary iron intake, NSAID use, and socioeconomic status, were identified, they were not adjusted for using multivariate analysis, which may introduce residual confounding. In addition, not all patients underwent endoscopic evaluation, and stool antigen testing was the primary diagnostic modality, which may have resulted in the underdetection of alternative GI causes of IDA, including bleeding lesions. Finally, important causes of IDA, such as gynecological blood loss, were not comprehensively assessed, which should be considered when interpreting the findings.

## Conclusions

This study demonstrated a significant association between *H. pylori *infection and the severity of IDA. Patients with *H. pylori *infection were more likely to present with moderate-to-severe anemia and exhibited significantly lower hemoglobin, serum ferritin, serum iron, and MCV levels, along with higher TIBC values compared to non-infected individuals. In addition, low dietary iron intake, gastrointestinal symptoms, and NSAID use were significantly associated with *H. pylori* infection, highlighting the multifactorial nature of IDA and the potential role of chronic infection in disrupting iron metabolism and worsening anemia severity. These findings suggest that *H. pylori* infection should be considered an important underlying factor in patients with unexplained or persistent IDA, particularly those with moderate-to-severe disease. Incorporating *H. pylori *screening into the routine evaluation of IDA may facilitate earlier diagnosis and more targeted management, potentially improving hematological outcomes. Future multicenter prospective studies are warranted to further elucidate the mechanisms linking *H. pylori *infection with iron deficiency and to assess the long-term impact of eradication therapy on anemia recovery and prevention.
